# Household Hints and Recipes

**Published:** 1883-10

**Authors:** 


### Household Hints & Recipes.

RENOVATING OLD OIL PAINTINGS.—In cleansing old paintings that have become dingy with soot and coal dust, substances are frequently employed that injure the painting by acting on the lighter and more delicate tints and shades.

Von Bibra has discovered a method which, according to Wieck's *Gewerbe Zeitung*, is both safe and rapid.

The painting is first removed from the frame, and the dust and smoke brushed off with a pencil or feather. After this it is washed with a sponge dipped in well water. It is next covered with a thick layer of soap; shaving soap is the best for the purpose, because it remains moist and does not dry on. After the soap has been on eight or ten minutes it is all washed off with a strong brush or pencil, adding a little water if necessary. The soap that still adheres is rinsed off sufficiently with water, and the picture left to dry.

When completely dry it is further cleansed with nitro-benzol. This chemical preparation is also known as nitro-benzine, artificial oil of bitter almonds, essence of mirbane, and is a yellowish, oily (*very poisonous*) liquid, with a powerful smell of bitter almonds. It is formed when coal tar benzol is mixed with fuming or concentrated nitric acid under suitable precautions. The nitro-benzol is poured into a dish or soup plate, and a clean linen rag dipped in it, and passed over the painting. This quickly removes all the inherent dirt. This linen rag must be frequently exchanged for a clean one. When the rag remains clean after going over it repeatedly, the cleansing is finished.

If the colors look dull after going over it the last time and letting it dry, it is given a thin coat of the finest olive oil, and after a while must be varnished with a good, quickly drying varnish.

It is claimed that the dirtiest oil paintings, when cleansed as above described, acquire their original colors and freshness.

---

A SUBSTITUTE FOR STAMPING PAD.—A. Vomacka gives, in a Bohemian journal called *Rundschau*, the following directions for making a color-mass for use with a stamp, to take the place of ink and pad:

Thirty or forty parts of crude anhydrous glycerine are saturated warm with any desirable blue, red, green, or black soluble aniline or eosine dye. Ten parts of fine glue are softened in water for twenty-four hours, the water drained off, and the swollen glue quickly dried between linen cloths and then melted on a water bath in the glycerine, the larger or smaller quantity being taken according to the kind of glue.

The water that is introduced in the swollen glue is evaporated without too much stirring, which would cause air bubbles and make it foamy. It is then cast in tin boxes.

If some air bubbles are noticed after it has been standing awhile, they can be removed by scraping the surface with a stiff card.

The uniform mass of glue and color represents the stamping pad when it is saturated with ink. But instead of requiring the addition of ink put on from time to time, it is always ready to use, saves ink, and saves the stamp from wear. The mass is elastic, so that the stamp can be pressed down quite hard on it. If this mass should be injured accidentally, it is only necessary to simply warm it and let it cool again. If it gets hard on the surface from not being used for a long time, and hence gives an imperfect impression, it is only necessary, to restore it, to wash it off carefully with a sponge dipped in warm water, or vinegar and water.

This stamping pad is best adapted to rubber stamps, but it can also be employed for a metal stamp if the surface of the latter is roughened with sand-paper or emery to make it take the ink.

---

THE SMOKE TEST FOR DRAINS.—This test is being much used in both England and Scotland, and is very satisfactory. It is applied by a small machine with powerful fanners, which blow the smoke of ignited cotton-waste, saturated with oil, into the drainage system, and in due time the smoke issues from all defective points and imperfect traps, showing clearly where the fault lies.

---

REMOVAL OF ENCAUSTIC LETTERS.—The signatures, letters, numbers, etc., upon porcelain vessels may be removed, according to E. Weymar, without injury to the glazing, by protracted polishing with a piece of pumice-stone moistened with concentrated hydrochloric acid. The removal is facilitated by previously exposing the signatures to the vapors of hydrochloric acid.—*Pharm. Zeit.*

